# Supplementary material for: The Donnan potential revealed
Source: Nat Commun. 2022 Oct 6;13:5880. doi: 10.1038/s41467-022-33592-3 (PMC9537300; doi:10.1038/s41467-022-33592-3)
Supplement: Supplementary file 1 — Supplementary Information [file 41467_2022_33592_MOESM1_ESM.pdf]

Supplementary Information for

'The Donnan Potential Revealed'

Pinar Aydogan Gokturk[1], Rahul Sujanani[2], Jin Qian[1,3], Ye Wang[1,3], Lynn Katz[4], Benny D. Freeman[2] and Ethan J. Crumlin[1,3*]

[1] Advanced Light Source, Lawrence Berkeley National Laboratory, Berkeley, CA 94720, United States

[2] McKetta Department of Chemical Engineering, The University of Texas at Austin, Austin, Texas 78712, United States

[3]Chemical Sciences Division, Lawrence Berkeley National Laboratory, Berkeley, California 94720, United States

[4]Department of Civil, Architectural, and Environmental Engineering, The University of Texas at Austin, Austin, Texas 78712, United States

Supplementary Figures

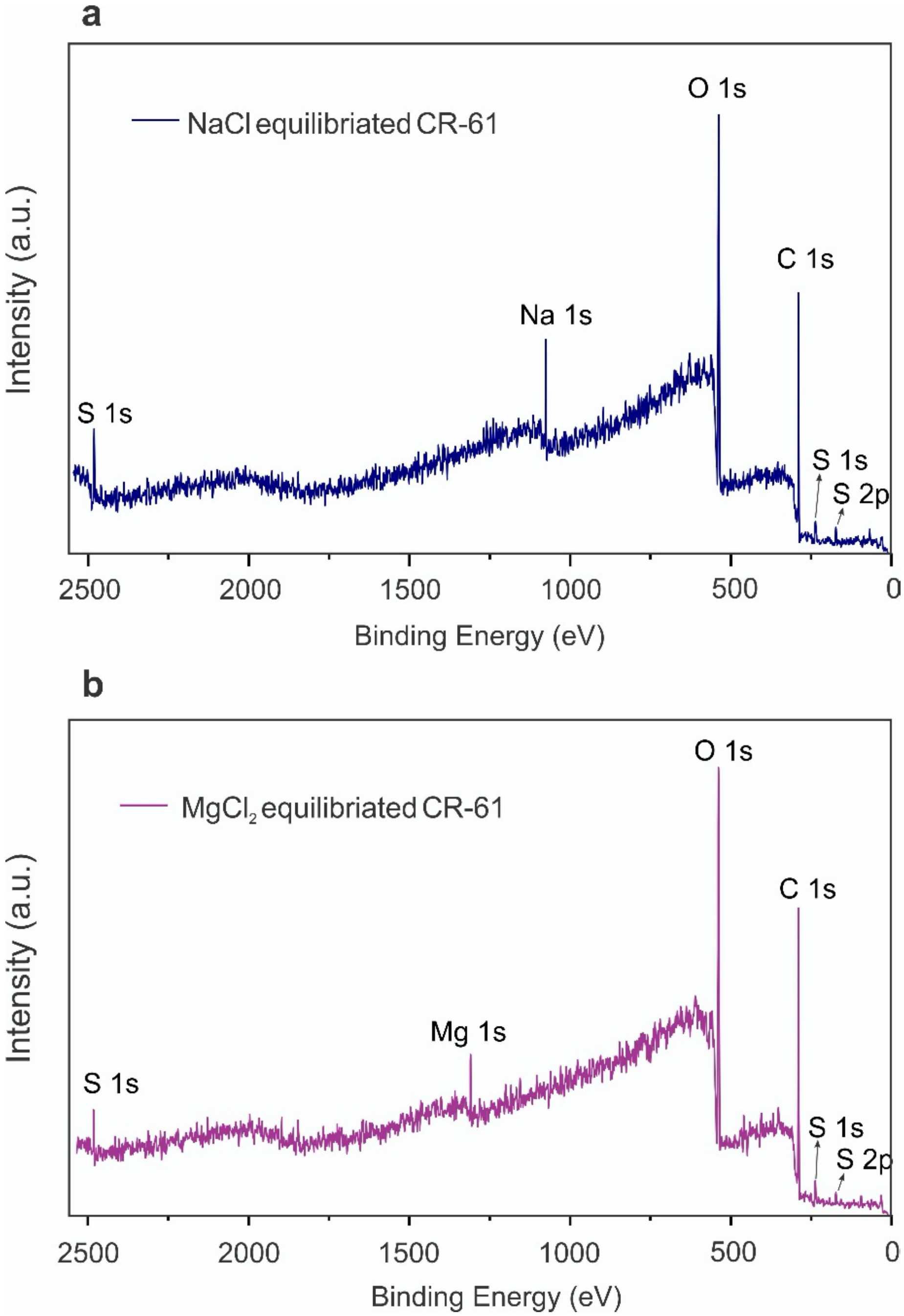

Supplementary Fig. 1. Survey spectra of membrane/solution/vapor system during the *in situ* tender-APXPS measurements for **a,** $NaCl_{(sol)}$ equilibrated CR-61 and **b,** $MgCl_{2\,(sol)}$ equilibrated CR-61 membrane. No other chemistry was observed in the system.

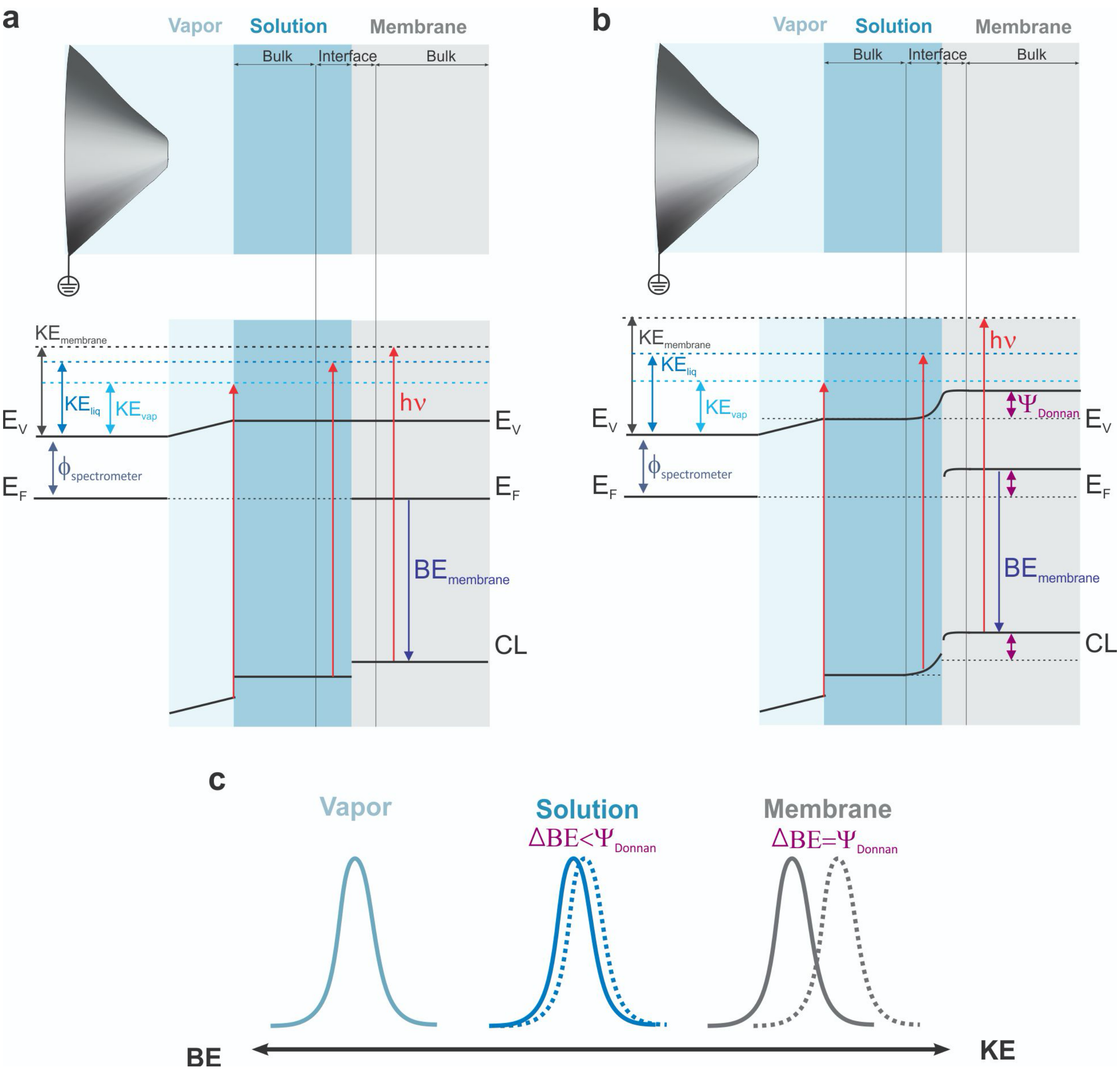

Supplementary Fig. 2. Energy level diagram of a membrane/solution/vapor system during the *operando* tender-APXPS measurements **a,** when there is no concentration difference between counter-ion species in the solution and the membrane phase (i.e., Ψ_{Donnan}= 0 V) and **b,** when there is a concentration gradient between the solution and membrane phase (i.e., concentration of counter-ions in solution is lower than the number of fixed ions in the membrane). **c,** Corresponding binding energy shifts in photoelectron spectra originating from each component of the system. Solid lines represent peaks under the potential gradient, and dashed lines indicate peaks when there

is no concentration difference between counter-ion species in the solution and the membrane phase (electrical potential is equal to 0 V). The electrical potential developed on the membrane effectively shifts all energy levels up (with negative potential) relative to the grounded analyzer. The measured binding energy of membrane related core level photoelectrons shows a shift that is related to the Donnan potential (Ψ_{Donnan}) on the surface by $\Delta BE = \Psi_{\text{Donnan}}$ eV (hν: incoming X-ray energy, E_F: Fermi level, E_v: vacuum level, CL: core level, $\Phi_{\text{spectrometer}}$: spectrometer work function, KE: photoelectron kinetic energy, BE: binding energy).

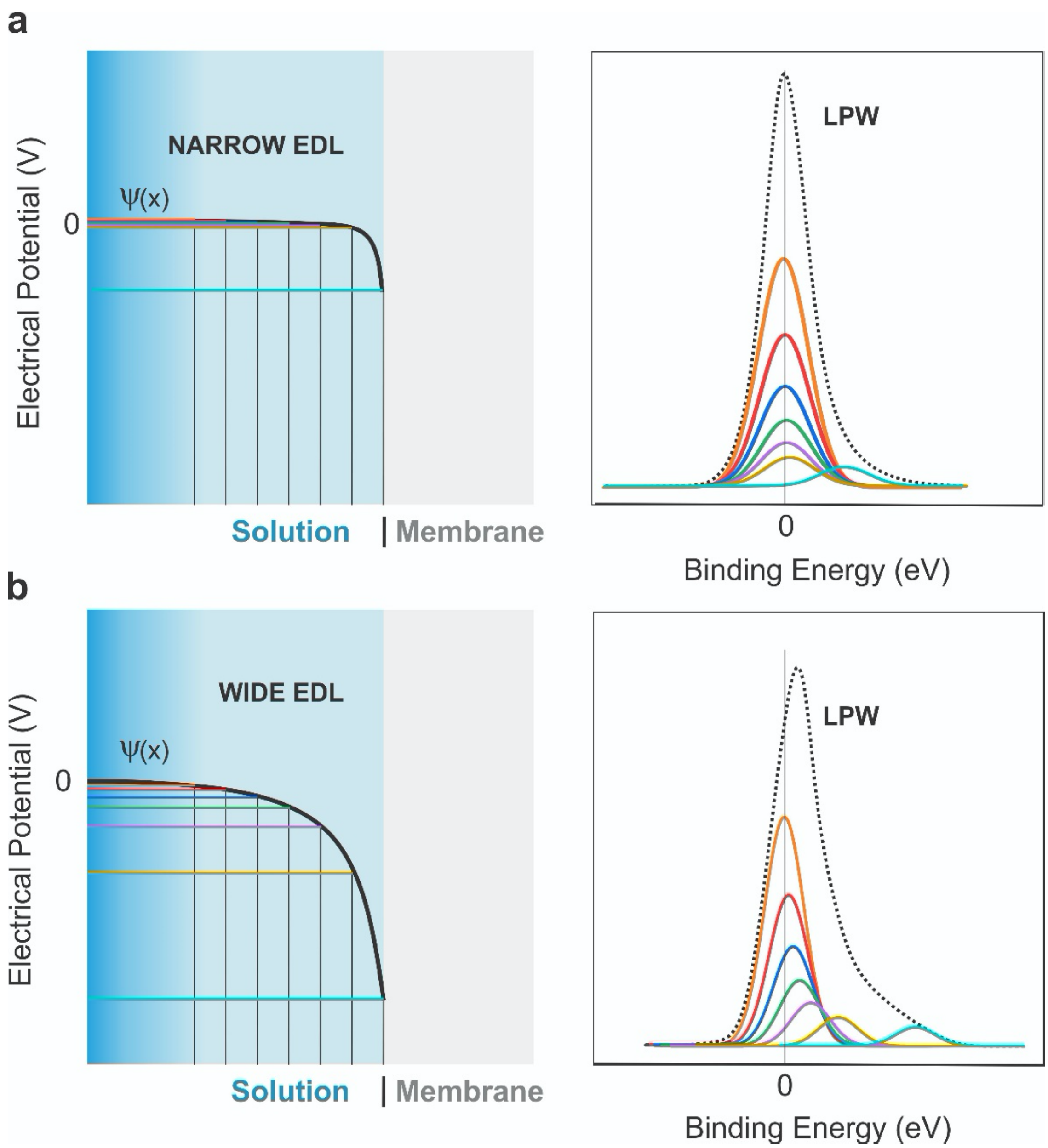

Supplementary Fig. 3 Schematic of the effect of EDL thickness on observed spectral BE shift and broadening of the liquid phase related core levels for **a,** a narrow and **b,** a wide EDL. EDL: electrochemical double layer, LPW: liquid phase water.

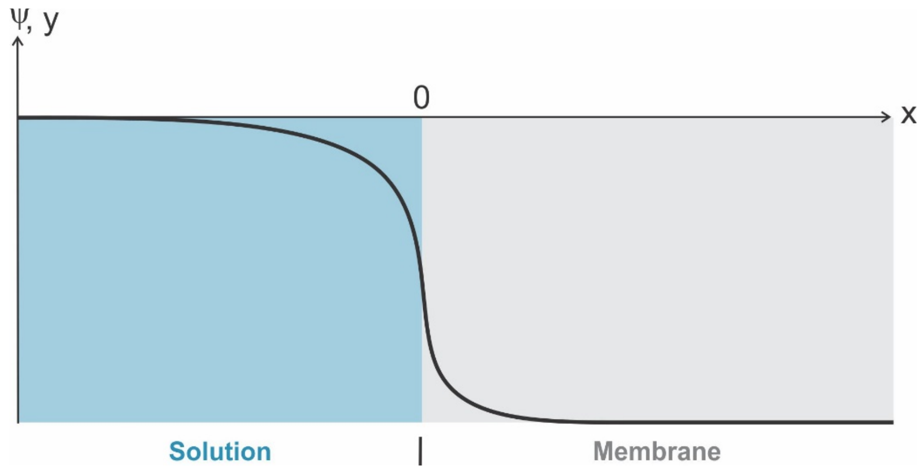

Supplementary Fig. 4. Schematic representation of the electrical potential distribution at the solution/membrane interface as a function of distance x.

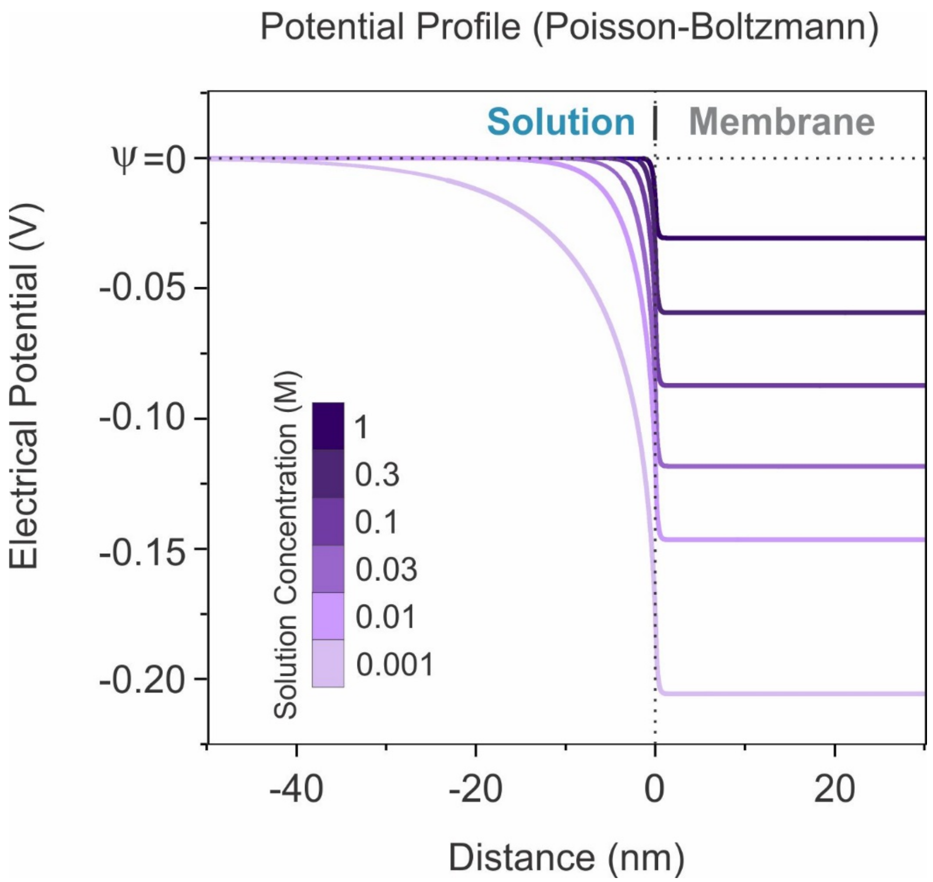

Supplementary Fig. 5. Simulated potential distribution at position (x) near the CR-61 membrane/solution interface.

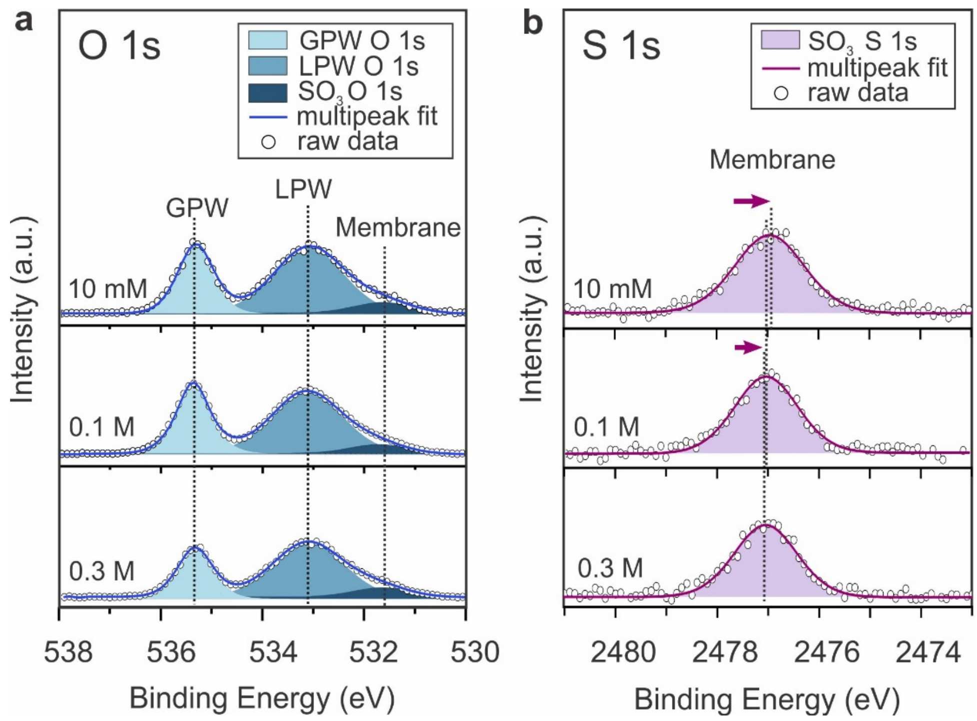

Supplementary Fig. 6. Experimental measurements of spectral binding energy shifts of sulfonate group with respect to solution-related core level peaks. Representative **a,** O *1s* and **b,** S *1s* core level spectra collected from CR-61 membranes equilibrated with the three other concentrations of NaCl solutions that are not presented in the main text. The binding energy is calibrated using bulk liquid phase water (LPW). The effect of the double layer on the binding energy position of the LPW peak is considered and corrections are made during the calibration process. (Details of the energy calibration process are given in **Supplementary Note 4**.) Circles represent the raw experimental data, and lines indicate the sum of fits. The binding energy values of fitted core level components for other individual analysis positions are provided in **Supplementary Table 6**.

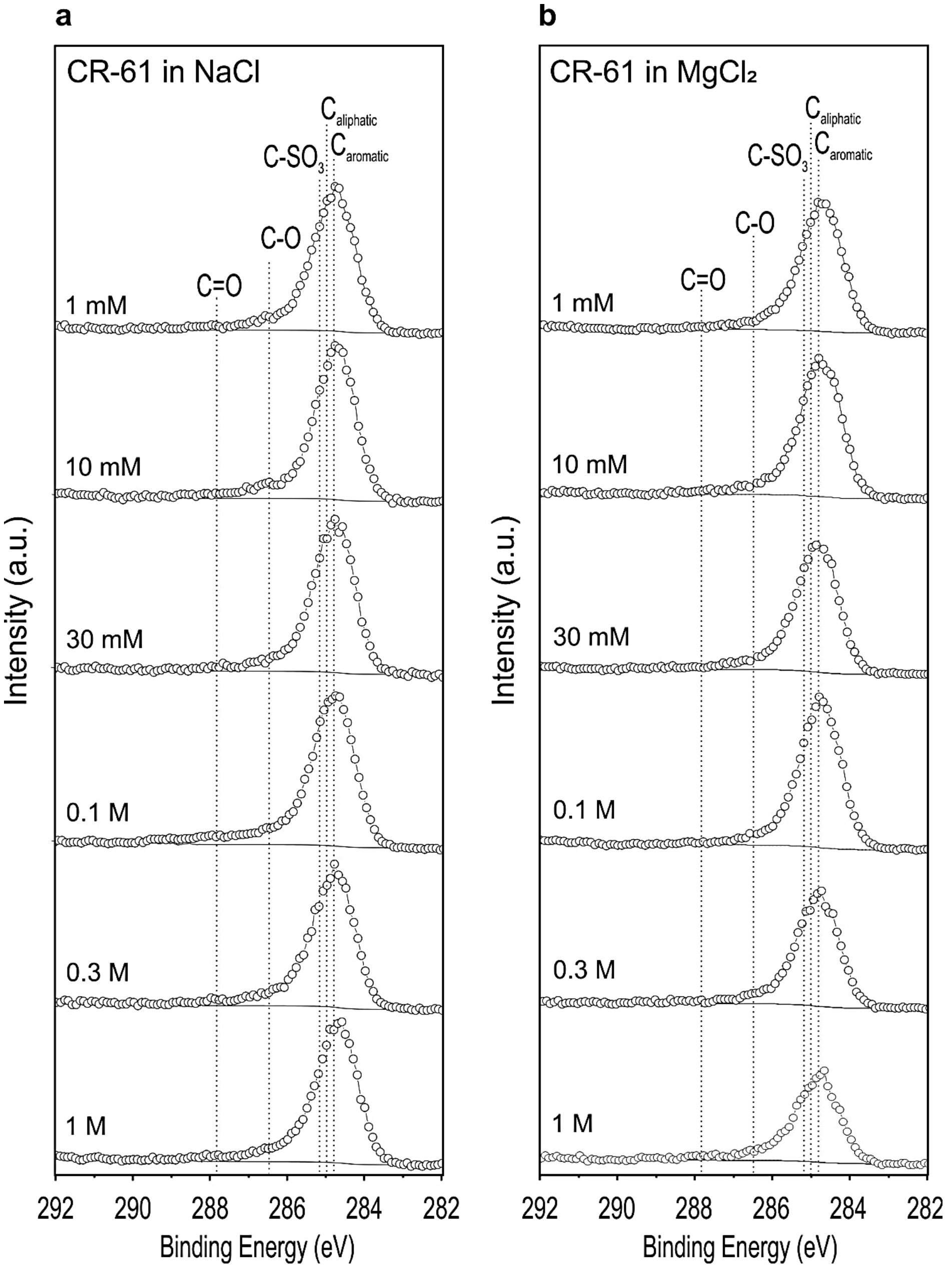

Supplementary Fig. 7 Representative C *1s* core level spectra collected from CR-61 membranes equilibrated with various concentrations of **a,** NaCl and **b,** $MgCl_2$ solution. The binding energy is calibrated by adjusting the aliphatic C to 285.0 eV. Circles represent the raw experimental data. Vertical dashed lines indicate the binding energy of possible individual chemical carbon contributions. Shirley background is included to be a visual guide for better capturing the relative amounts of each chemistry. All binding energies are obtained from ref 6.

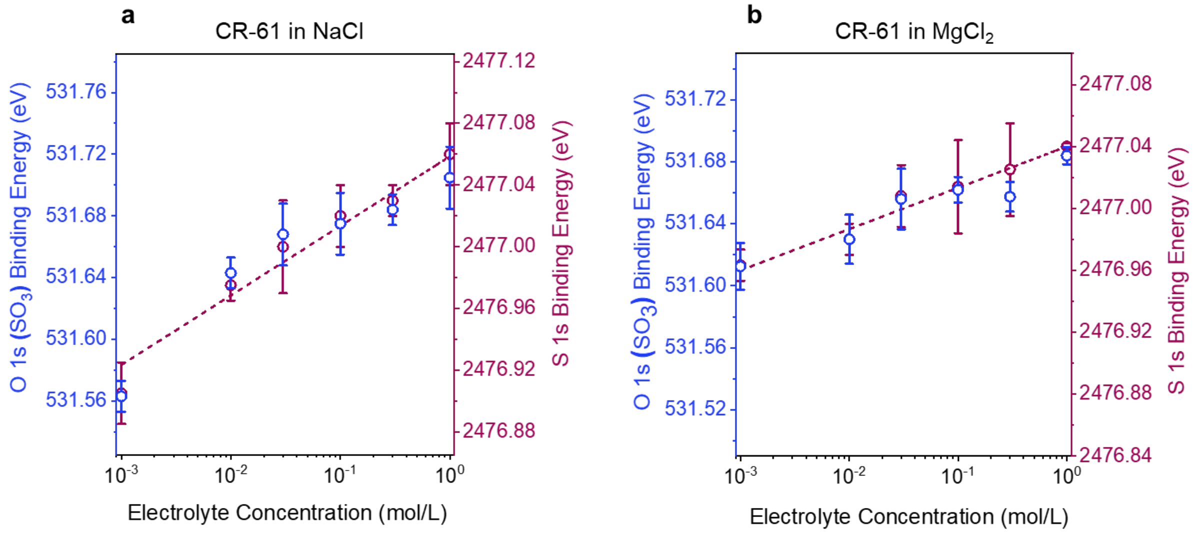

Supplementary Fig. 8. Comparison of experimentally measured binding energy shifts in membrane fixed ion related core levels as a function of external **a,** NaCl and **b,** $MgCl_2$ solution concentration.

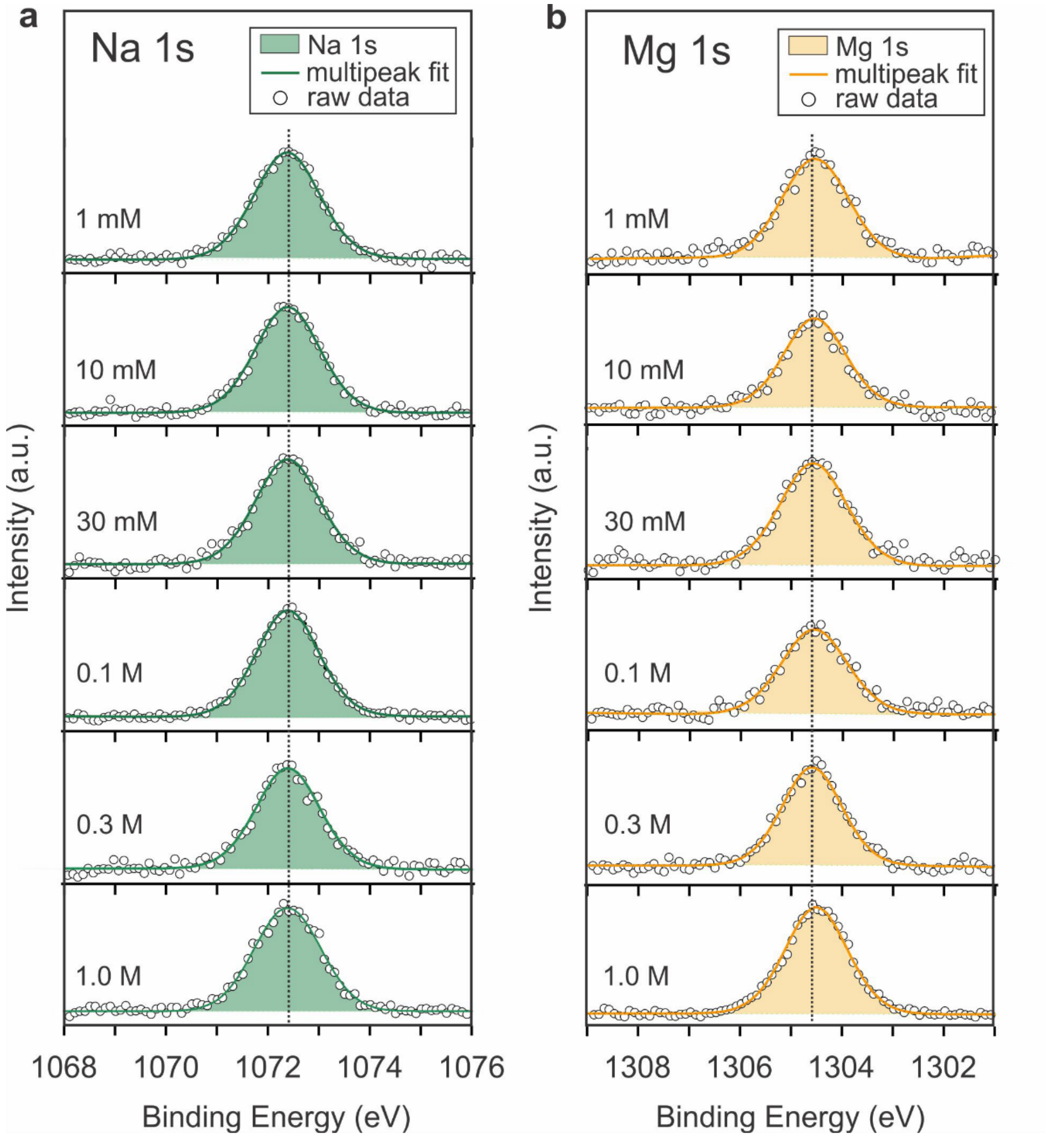

Supplementary Fig. 9. Representative **a**, Na *1s* and **b**, Mg *1s* core level spectra collected from CR-61 membranes equilibrated with various concentrations of NaCl and $MgCl_2$ solutions. The binding energy is calibrated using bulk liquid phase water (LPW). Circles represent the raw experimental data, and lines indicate the sum of fits. The binding energy value of fitted core level components for each individual analysis position are provided in **Supplementary Table 6**.

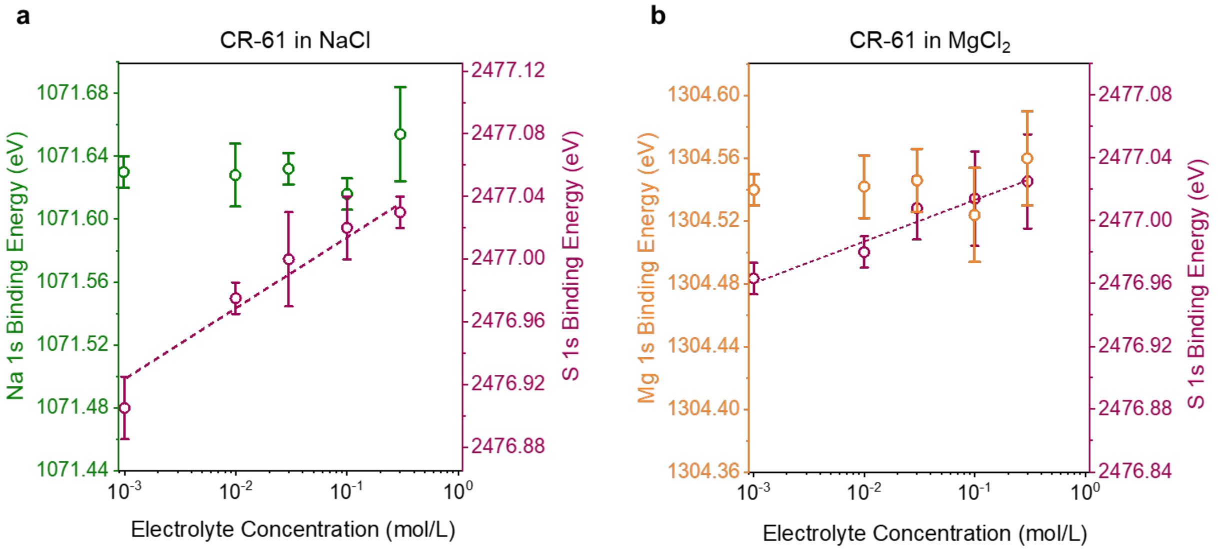

Supplementary Fig. 10. Comparison of experimentally measured binding energy shifts in counterion and membrane specific core levels as a function of external **a,** NaCl and **b,** $MgCl_2$ solution concentration.

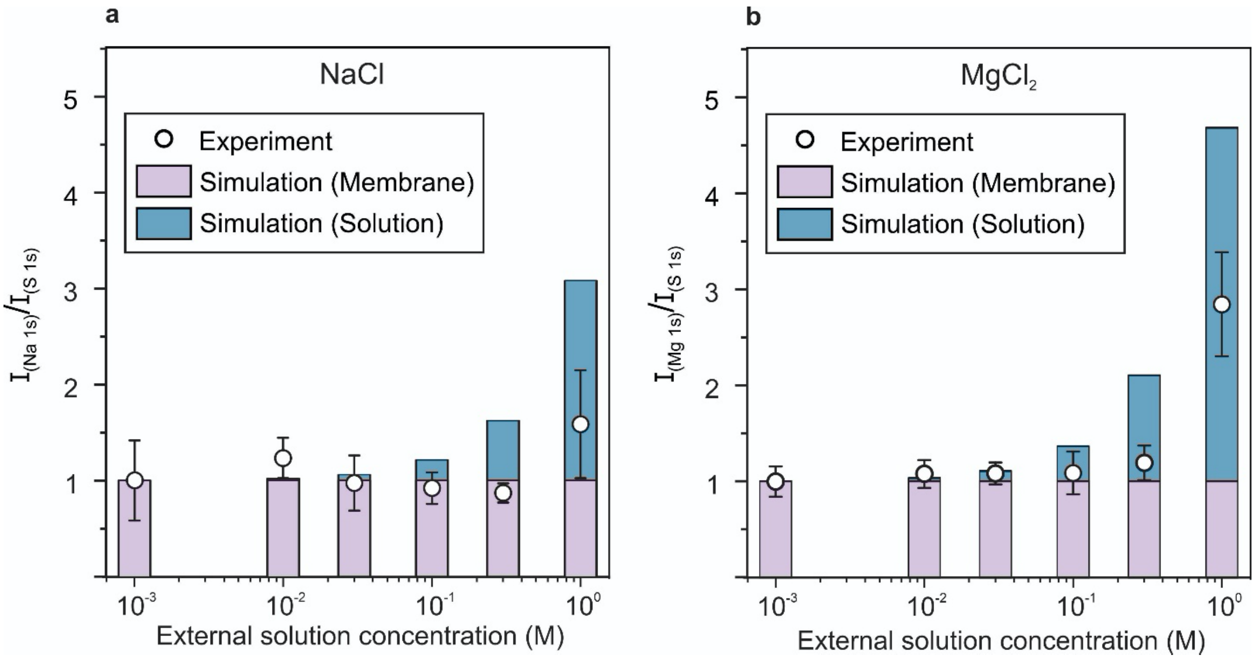

Supplementary Fig. 11. Experimental trends and simulated ratio of **a,** Na *1s* and **b,** Mg *1s* peak areas to S *1s* as a function of external salt solution concentration. Purple bars represents the simulated intensity contribution of membrane, while the blue bars represent the contribution from the solution layer. Error bars shows the experimental uncertainty, determined from the standard deviation of repeated measurements of four or more different positions in each case.

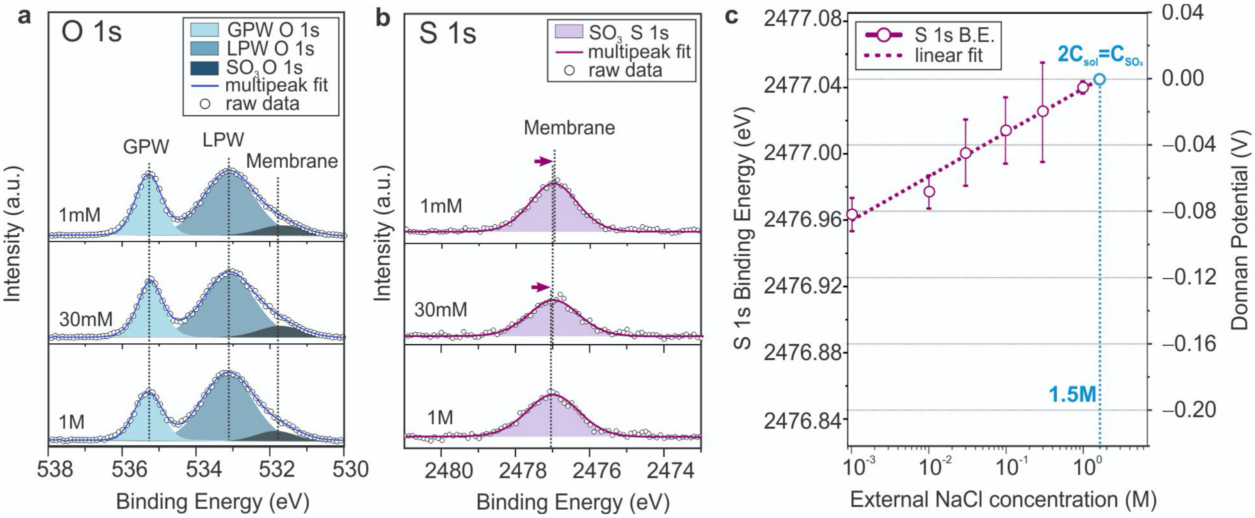

Supplementary Fig. 12. Experimental measurements of the Donnan potential from spectral binding energy shifts of sulfonate groups with respect to solution related core level peaks. Representative **a,** O *1s* and **b,** S *1s* core level spectra collected from CR-61 membranes equilibrated with different concentrations of $MgCl_2$. Circles represent raw experimental data, and lines indicate the sum of fits. Representative O *1s* and S *1s* core level spectra collected from CR-61 membranes equilibrated with three other concentrations of $MgCl_2$ solutions are presented in **Supplementary Fig 13. c,** Averaged S *1s* binding energy and corresponding Donnan potential values as a function of the external solution concentration. Error bars represent experimental uncertainty, determined from the standard deviation of repeated measurements of four or more different positions in each case. The binding energy values of fitted core level components for each individual analysis position are provided in **Supplementary Table 6**. The dashed line represents a linear best fit. The fitted line was extrapolated to the equivalent concentration of counter-ions (1.5 M for $MgCl_2$) where the Donnan potential approaches to 0 V.

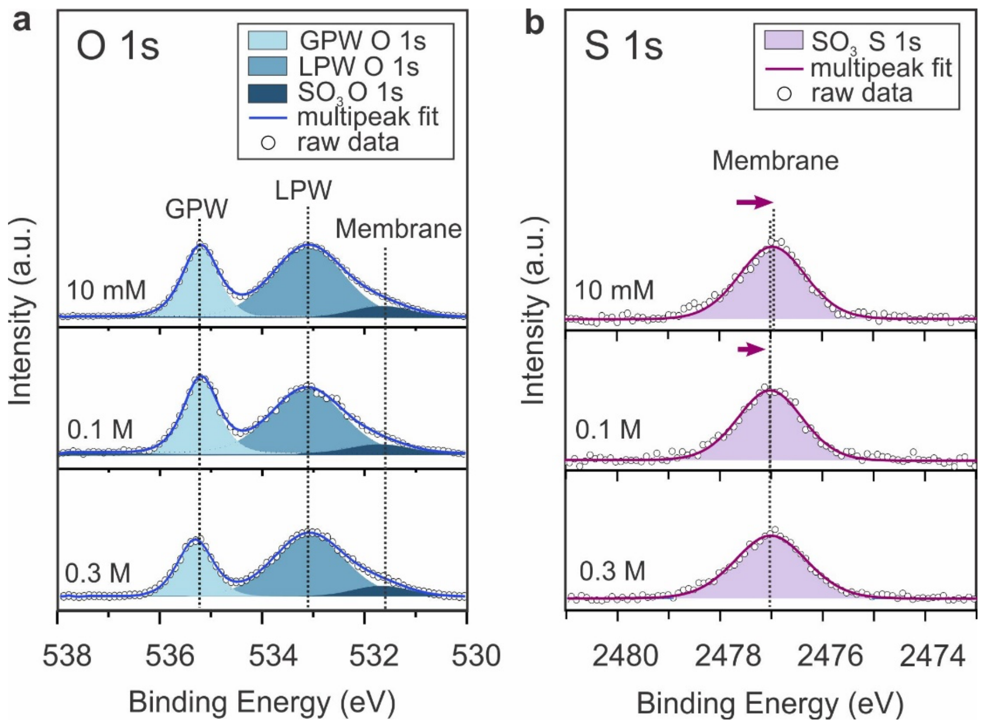

Supplementary Fig. 13. Experimental measurements of spectral binding energy shifts of sulfonate group with respect to solution-related core levels. Representative **a,** O *1s* and **b,** S *1s* core level spectra collected from CR-61 membranes equilibrated with other three concentrations of $MgCl_2$ solutions (not presented in the **Supplementary Fig. 12**). The binding energy is calibrated using bulk liquid phase water (LPW). Circles represent the raw experimental data, and lines indicate the sum of fits. The binding energy value of fitted core level components for each individual analysis position are provided in **Supplementary Table 6**.

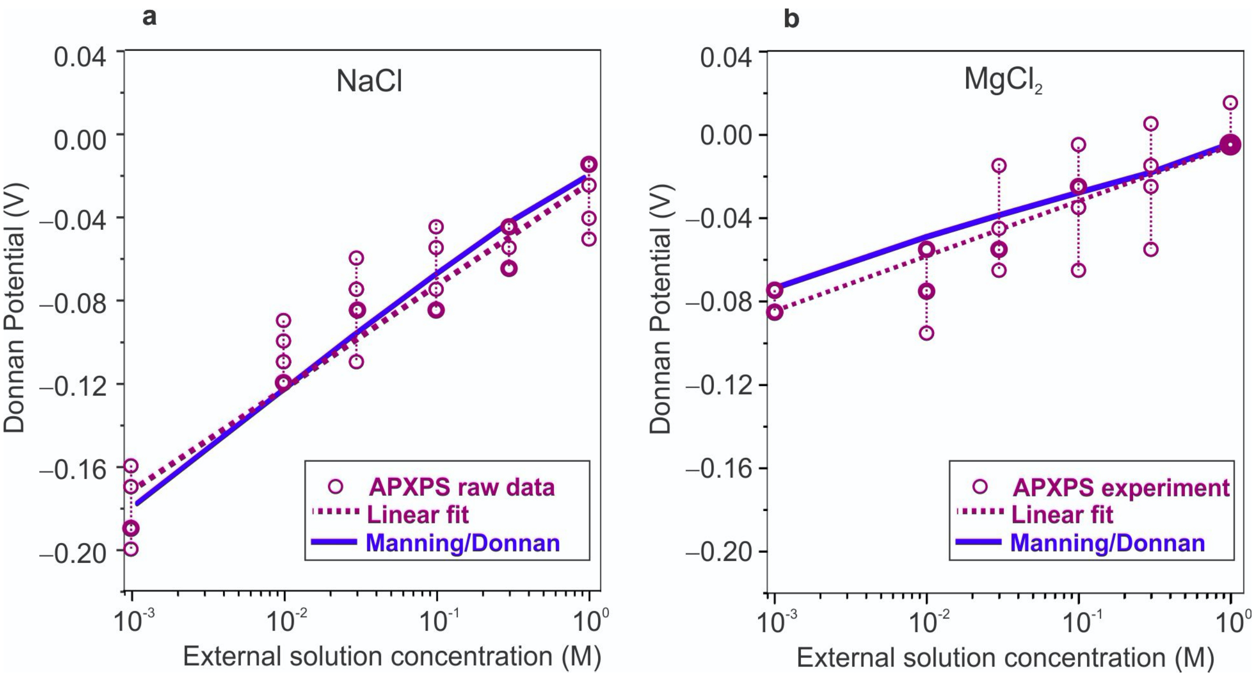

Supplementary Fig. 14. Comparison between the experimental and predicted Donnan potential trends, values, and the effect of counter-ion valence on Donnan potential. Comparison of experimentally measured Donnan potential values obtained from binding energy shifts in membrane related core levels with the Manning/Donnan model predictions as a function of external **a,** NaCl and **b,** $MgCl_2$ solution concentration. Dashed lines are the linear fits of APXPS experimental data averages for external NaCl and $MgCl_2$ solutions with slopes of 0.049$\pm$0.004 and 0.026$\pm$0.002, respectively. The plots are the same as those in **Fig. 3**; we have included the raw data here as well to facilitate the statistical significance discussion.

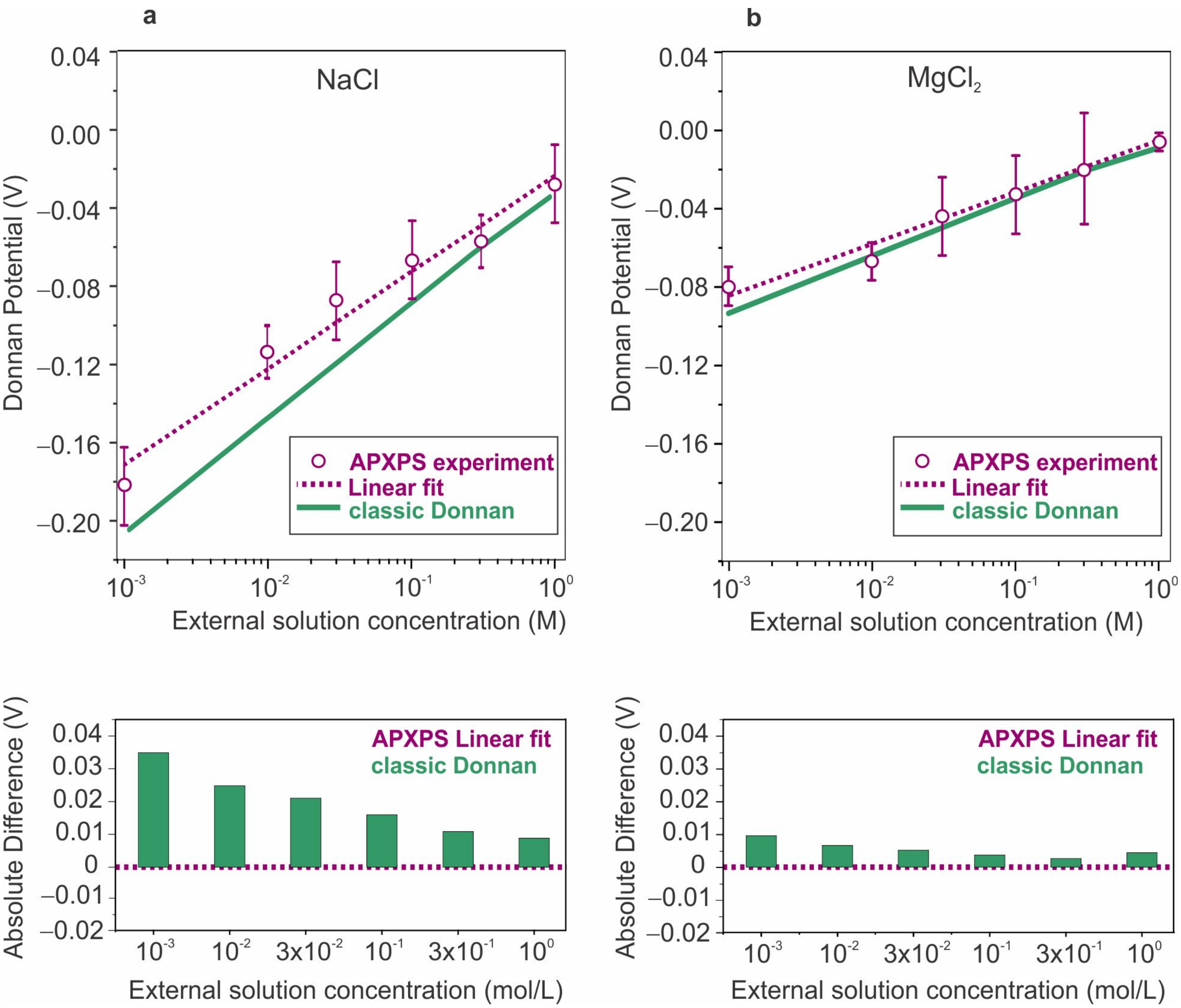

Supplementary Fig. 15. Comparison between the experimental and predicted Donnan potential trends, values, and the effect of counter-ion valence on Donnan potential. Comparison of experimentally measured Donnan potential values obtained from binding energy shifts in membrane related core levels with the classic Donnan model predictions as a function of external **a,** NaCl and **b,** $MgCl_2$ solution concentration. Error bars represent experimental uncertainty and are the standard deviation of repeated measurements of a minimum of five different positions in each case. Dashed lines are the linear fits of APXPS experimental data for external NaCl and $MgCl_2$ solutions with slopes of 0.049 and 0.026, respectively. (Details on Donnan potential calculations using the classic Donnan models appear in **Supplementary Note**

7). The absolute difference between the linear fit of the experimental data and predictions from the analytical model is provided to better capture the comparison between theoretical model and experimental result for each electrolyte concentration.

Supplementary Tables

Supplementary Table 1. Properties of CR-61 membranes used in this study.

Chemical Structure	Thickness[a]	Reported IEC[b]	Fixed-ion concentration[c]	Water uptake[d]
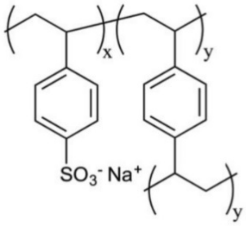	0.06	2.2 (minimum)	3.21 ± 0.08	0.84 ± 0.01

[a][cm]
[b][meq/g (dry polymer)]. IEC: ion-exchange capacity. Value was obtained from specification sheets provided directly by the manufacturer (Suez).
[c][mol fixed charge groups/L (water sorbed)]. Estimated from the counter-ion concentration in the membrane at an external NaCl concentration of 0.01 M [1].
[d][g (water)/g (dry polymer)]. This value refers to the equilibrium water content of Na^+ counter-ion form membranes equilibrated in ultrapure DI water. It represents the water uptake in only the active ion exchange polymer phase in the composite CR-61 membrane (i.e., the contribution of the fabric backing has been removed as detailed elsewhere[2]).

Supplementary Table 2. Parameters used to estimate the liquid layer thickness on the CR-61 membrane.

$n^{\mathrm{m}}_{SO_3^-}$	λ_{memb}	$\lambda_{solution}$	$n^{\mathrm{m}}_{\mathrm{w}}$ (for NaCl)	$n^{\mathrm{m}}_{\mathrm{w}}$ (for $MgCl_2$)	$n^{\mathrm{s}}_{\mathrm{w}}$
1.03 x 10^{22} /dm^3	8.3 nm	9.5 nm	2.06 x 10^{22} /dm^3	3.09 x 10^{22} /dm^3	3.35 x 10^{22} /dm^3

Supplementary Table 3. Relative intensities of the solution and membrane related O *1s* (LPW and SO_3^- components) XPS peaks of NaCl equilibrated CR-61 collected from five different positions for each electrolyte concentration.

	P #1	P #2	P #3	P #4	P #5
**1 mM NaCl**	8.2	7.4	7.6	9.3	9.5
**10 mM NaCl**	9.8	8.2	7.2	7.2	7.2
**30 mM NaCl**	7.5	7.9	7.1	7.6	7.0
**0.1 M NaCl**	8.4	7.3	8.9	7.9	7.6
**0.3 M NaCl**	7.0	9.6	7.2	7.3	7.5
**1 M NaCl**	7.4	7.3	8.2	9.3	8.5

Supplementary Table 4. Relative intensities of the solution and membrane related O *1s* (LPW and SO_3^- components) XPS peaks of $MgCl_2$ equilibrated CR-61 system collected from five different positions for each electrolyte concentration.

	P #1	P #2	P #3	P #4	P #5
**1 mM $MgCl_2$l**	8.5	7.8	8.3	7.8	N/A
**10 mM $MgCl_2$**	7.2	7.0	7.0	7.4	8.3
**30 mM $MgCl_2$**	9.8	11.6	10.1	9.5	8.4
**0.1 M $MgCl_2$**	9.7	9.0	8.6	7.5	8.5
**0.3 M $MgCl_2$**	11.6	10.8	11.0	12.4	N/A
**1 M $MgCl_2$**	8.2	10.8	12.3	10.9	11.8

Supplementary Table 5. Peak fitting parameters and constraints used for this study. Peak assignments were made by assuming the minimum number of peaks necessary to fit spectral features. Binding energies (BE – maxima and minima reported) and full-width-half-maxima (FWHM) agree well with values reported in literature[3,6,7].

	**O *1s*-GPW**	**O *1s*-LPW**	**O *1s*- SO_3**	**S *1s*-SO_3**
**Line Shape**	GL(50-70)	GL(30)	GL(30)	GL(30)
**FWHM**	0.7-0.9	1.6-1.9	1.2-1.4	1.4-1.7
**Binding Energy (eV)[a]**	535.20-535.40	533.11	531.55-531.75	-

[a] Binding energy values of fitted core level peaks are provided in **Supplementary Table 6.**

Supplementary Table 6. Binding energy results of fitted core level peaks of membrane/solution system collected from four or five different positions for each electrolyte concentration.

**Name**	**P #1 B.E. (eV)**	**P #2 B.E. (eV)**	**P #3 B.E. (eV)**	**P #4 B.E. (eV)**	**P #5 B.E. (eV)**
**CR61- 1 mM NaCl**					
**O *1s*-LPW**	533.11	533.11	533.11	533.11	533.11
**O *1s*-GPW**	535.28	535.30	535.25	535.28	535.33
**O *1s*- SO_3**	531.59	531.62	531.60	531.58	531.60
**S *1s*-SO_3**	2476.95	2476.96	2476.95	2476.92	2476.93
**Na *1s***	1071.62	1071.64	1071.63	1071.62	1071.64
**CR61- 10 mM NaCl**					
**O *1s*-LPW**	533.11	533.11	533.11	533.11	533.11
**O *1s*-GPW**	535.30	535.26	535.31	535.30	535.35
**O *1s*- SO_3**	531.64	531.65	531.66	531.66	531.63
**S *1s*-SO_3**	2477.00	2476.99	2476.98	2476.98	2476.97
**Na *1s***	1071.62	1071.62	1071.62	1071.66	1071.62
**CR61- 30mM NaCl**					
**O *1s*-LPW**	533.11	533.11	533.11	533.11	533.11

**O *1s*-GPW**	535.22	535.30	535.25	535.24	535.29
**O *1s*- SO_3**	531.65	531.66	531.69	531.68	531.66
**S *1s*-SO_3**	2476.98	2477.01	2477.01	2477.03	2477.00
**Na *1s***	1071.62	1071.64	1071.64	1071.64	1071.62
**CR61- 0.1 M NaCl**					
**O *1s*-LPW**	533.11	533.11	533.11	533.11	533.11
**O *1s*-GPW**	535.37	535.34	535.32	535.31	535.33
**O *1s*- SO_3**	531.70	531.65	531.67	531.68	531.64
**S *1s*-SO_3**	2477.00	2477.01	2477.00	2477.04	2477.03
**Na *1s***	1071.61	1071.60	1071.62	1071.63	1071.62
**CR61- 0.3 M NaCl**					
**O *1s*-LPW**	533.11	533.11	533.11	533.11	533.11
**O *1s*-GPW**	535.26	535.30	535.35	535.35	535.23
**O *1s*- SO_3**	531.72	531.67	531.67	531.69	531.69
**S *1s*-SO_3**	2477.04	2477.03	2477.02	2477.02	2477.04
**Na *1s***	1071.68	1071.68	1071.64	1071.62	1071.65

**CR61- 1 M NaCl**					
**O *1s*-LPW**	533.11	533.11	533.11	533.11	533.11
**O *1s*-GPW**	535.30	535.33	535.30	535.34	535.29
**O *1s*- SO_3**	531.70	531.70	531.71	531.68	531.73
**S *1s*-SO_3**	2477.07	2477.07	2477.05	2477.03	2477.06
**Na *1s***	1071.61	1071.68	1071.63	1071.63	1071.61
**CR61- 1 mM $MgCl_2$**					
**O *1s*-LPW**	533.11	533.11	533.11	533.11	N/A
**O *1s*-GPW**	535.26	535.15	535.21	535.24	N/A
**O *1s*- SO_3**	531.60	531.60	531.63	531.62	N/A
**S *1s*-SO_3**	2476.96	2476.96	2476.97	2476.97	N/A
**Mg *1s***	1304.53	1304.54	1304.54	1304.55	N/A
**CR61- 10 mM $MgCl_2$**					
**O *1s*-LPW**	533.11	533.11	533.11	533.11	533.11
**O *1s*-GPW**	535.23	535.19	535.20	535.24	535.18
**O *1s*- SO_3**	531.64	531.65	531.63	531.61	531.63

**S *1s*-SO_3**	2476.97	2476.99	2476.96	2476.99	2476.99
**Mg *1s***	1304.56	1304.52	1304.56	1304.53	1304.54
**CR61- 30 mM $MgCl_2$**					
**O *1s*-LPW**	533.11	533.11	533.11	533.11	533.11
**O *1s*-GPW**	535.25	535.28	535.19	535.22	535.23
**O *1s*- SO_3**	531.67	531.67	531.64	531.67	531.63
**S *1s*-SO_3**	2476.99	2477.00	2477.03	2476.98	2476.99
**Mg *1s***	1304.54	1304.53	1304.53	1304.57	1304.56
**CR61- 0.1 M $MgCl_2$**					
**O *1s*-LPW**	533.11	533.11	533.11	533.11	533.11
**O *1s*-GPW**	535.24	535.16	535.21	535.19	535.20
**O *1s*-SO_3**	531.67	531.66	531.65	531.67	531.66
**S *1s*-SO_3**	2477.01	2477.04	2477.02	2476.98	2477.01
**Mg *1s***	1304.56	1304.51	1304.50	1304.51	1304.54
**CR61- 0.3 M $MgCl_2$**					
**O *1s*-LPW**	533.11	533.11	533.11	533.11	N/A

**O *1s*-GPW**	535.14	535.11	535.16	535.17	N/A
**O *1s*-SO_3**	531.65	531.67	531.65	531.66	N/A
**S *1s*-SO_3**	2477.05	2477.03	2477.02	2476.99	N/A
**Mg *1s***	1304.52	1304.56	1304.59	1304.57	N/A
**CR61- 1 M $MgCl_2$**					
**O *1s*-LPW**	533.11	533.11	533.11	533.11	533.11
**O *1s*-GPW**	535.14	535.19	535.05	535.01	535.09
**O *1s*-SO_3**	531.68	531.68	531.70	531.69	531.68
**S *1s*-SO_3**	2477.04	2477.04	2477.04	2477.06	2477.04
**Mg *1s***	1304.50	1304.50	1304.48	1304.46	N/A

Supplementary Table 7. Estimated EDL thicknesses for six external NaCl concentrations**.**

Salt Concentration [mol/L]	EDL Thickness (nm)	
	**Debye Length**	**PB Equation**
0.001	9.6	40
0.01	3.0	13.5
0.03	1.8	7.5
0.1	1.0	3.7
0.3	0.6	1.7
1.0	0.3	0.8

Supplementary Table 8. Relative shifts in the binding energy of LPW obtained from simulations at six external NaCl concentrations.

NaCl Concentration [mol/L]	Binding Energy Shifts in LPW (eV)
0.001	-0.035
0.01	-0.005
0.03	-0.001*
0.1	>-0.001*
0.3	>-0.001*
1.0	>-0.001*

* below current experimental detection limits

Supplementary Table 9. Estimated IMFPs for a given core level in the aqueous salt solution and the membrane using the Tanuma-Powell-Penn (TPP-2M) algorithm and the modified Bethe equation[3,4].

Core level	λ_{memb}	$\lambda_{solution}$
Na *1s*	7.2 nm	9.2 nm
Mg *1s*	6.7 nm	8.6 nm
S *1s*	4.2 nm	5.3 nm

Supplementary Table 10 Apparent fit parameters and ANOVA results for the experimentally measured binding energy shifts in membrane related core levels of NaCl-equilibrated CR61.

<table>
<tr><td>PARAMETERS</td><td>Value</td><td>Standard Error</td><td>t-Value</td><td>Prob>|t|</td><td></td></tr>
<tr><td>Intercept</td><td>2477.06</td><td>0.007</td><td>331782.47</td><td>4.95151E-22</td><td></td></tr>
<tr><td>Slope</td><td>0.049</td><td>0.004</td><td>11.28</td><td>3.51475E-4</td><td></td></tr>
<tr><td>ANOVA</td><td>DF</td><td>Sum of Squares</td><td>Mean Square</td><td>F Value</td><td>Prob>F</td></tr>
<tr><td>Model</td><td>1</td><td>34.53004</td><td>34.53004</td><td>127.32879</td><td>3.51475E-4</td></tr>
<tr><td>Error</td><td>4</td><td>1.08475</td><td>0.27119</td><td></td><td></td></tr>
<tr><td>Total</td><td>5</td><td>35.61479</td><td></td><td></td><td></td></tr>
</table>

Supplementary Table 11 Apparent fit parameters and ANOVA results for the experimentally measured binding energy shifts in membrane related core levels of $MgCl_2$-equilibrated CR61.

**PARAMETERS**	Value	Standard Error	t-Value	Prob>|t|	
Intercept	2477.04	0.004	641282.80	3.54775E-23	
Slope	0.0261	0.002	11.24	3.57243E-4	
**ANOVA**	DF	Sum of Squares	Mean Square	F Value	Prob>F
Model	1	0.00395	0.00395	126.26996	3.57242E-4
Error	4	1.25244E-4	3.1311E-5		
Total	5	0.00408			

Supplementary Table 12. Predicted C_g^m values for CR-61 equilibrated with 0.001-1 M NaCl and $MgCl_2$ solutions using the classical Donnan model.

**Salt Concentration [mol/L]**	C_A^m **[mol/L sorbed water]**		**Predicted** C_g^m **[mol/L sorbed water]**	
	**NaCl**	$MgCl_2$	**NaCl**	$MgCl_2$
0.001	3.2	3.0	3.20	1.50
0.01	3.2	3.0	3.20	1.50
0.03	3.2	3.0	3.21	1.50
0.1	3.2	3.0	3.23	1.51
0.3	3.2	3.0	3.29	1.56
1.0	3.2	3.0	3.49	2.00

Supplementary Table 13. Predicted Ψ_{Donnan} values for CR-61 equilibrated with 0.001-1 M NaCl and $MgCl_2$ solutions using the classic Donnan model.

Salt Concentration [mol/L]	NaCl Equilibrated Ψ_{Donnan} [V]	$MgCl_2$ Equilibrated Ψ_{Donnan} [V]
0.001	−0.207	−0.0938
0.01	−0.148	−0.0643
0.03	−0.120	−0.0502
0.1	−0.0891	−0.0348
0.3	−0.0611	−0.0211
1.0	−0.0322	−0.0089

Supplementary Table 14. Values of C_g^m and γ_g^m at a given external NaCl salt concentration predicted from the Manning/Donnan model using a known C_A^m value.

Salt Concentration [mol/L]	C_A^m [mol/L sorbed water]	Predicted C_g^m [mol/L sorbed water]	Predicted γ_g^m [mol/L sorbed water]
0.001	3.2	3.2	0.331
0.01	3.2	3.2	0.331
0.03	3.2	3.2	0.332
0.1	3.2	3.2	0.334
0.3	3.2	3.3	0.348
1.0	3.2	3.6	0.423

Supplementary Table 15. Values of C_g^m and γ_g^m at a given external $MgCl_2$ salt concentration predicted from the Manning/Donnan model using a known C_A^m value.

Salt Concentration [mol/L]	C_A^m [mol/L sorbed water]	Predicted C_g^m [mol/L sorbed water]	Predicted γ_g^m [mol/L sorbed water]
0.001	3.0	1.5	0.166
0.01	3.0	1.5	0.167
0.03	3.0	1.5	0.169
0.1	3.0	1.5	0.181
0.3	3.0	1.6	0.220
1.0	3.0	2.0	0.382

Predicted values of Ψ_{Donnan} for CR-61 using the combined Manning/Donnan model are given in **Supplementary Table 16.**

Supplementary Table 16. Predicted Ψ_{Donnan} values for CR-61 equilibrated with 0.001-1 M NaCl and $MgCl_2$ solutions using the Manning/Donnan model and equation S8**,** where γ_g^m values were calculated using Manning's model, and γ_g^s values were estimated using the Pitzer model.

Salt Concentration [mol/L]	NaCl Equilibrated Ψ_{Donnan} [V]	$MgCl_2$ Equilibrated Ψ_{Donnan} [V]
0.001	−0.180	−0.0740
0.01	−0.123	−0.0493
0.03	−0.0958	−0.0389
0.1	−0.0672	−0.0280
0.3	−0.0424	−0.0181
1.0	−0.0196	−0.0042

Supplementary Notes

Supplementary Note 1.

Attenuation of photoelectron peaks and estimating the solution layer thickness

The main challenge of the membrane/liquid study with APXPS is related to the short inelastic mean free paths (IMFPs) of photoelectrons, the very same characteristic that makes the technique surface sensitive. In our experimental setup, membrane related core level photoelectrons (i.e., S *1s*, C *1s* and O *1s*-SO_3) are attenuated through both the liquid and the gas phase. Creating a liquid film that is sufficiently stable and robust to be representative of a realistic membrane/solution interface at equilibrium, but thin enough to allow signal detection from the interfacial region, is very important. Considering these factors, a liquid layer of appropriate thickness was formed on the membrane.

The thickness of the liquid layer on the CR-61 ion exchange membrane was estimated from the relative intensities of the solution and membrane related O *1s* (LPW and SO_3^- components) XPS peaks, taking into consideration the attenuations of measured membrane related O *1s* XPS intensities due to the liquid layer. The intensity of the O *1s* peak for the SO_3^- component underneath the liquid layer of thickness d is obtained by integrating over the exponential escape probability as follows:

$$I_{SO_3^-} = 3\, n^{\mathrm{m}}_{SO_3^-}\, \sigma^{\mathrm{m}}_{SO_3^-}\, \lambda_{memb}\, e^{-d/\lambda_{solution}} \quad \text{(S1)}$$

where $n^{\mathrm{m}}_{SO_3^-}$ is the number density of sulfonate groups, $\sigma^{\mathrm{m}}_{SO_3^-}$ is the photoionization cross-section of oxygen, and λ_{memb} and $\lambda_{solution}$ are the inelastic mean free paths (IMFP) in membrane and solution, respectively. Unlike the case for common solid samples, the liquid phase water (LPW)

O *1s* signal intensity in our experimental system arises from both the liquid layer and the sorbed water inside the membrane. The intensity of the O *1s* peak for LPW is:

$$I_{LPW} = n_{\mathrm{w}}^{\mathrm{m}}\ \sigma_{\mathrm{w}}^{\mathrm{m}}\ \lambda_{memb}\ e^{-d/\lambda_{solution}} + n_{\mathrm{w}}^{\mathrm{s}}\ \sigma_{\mathrm{w}}^{\mathrm{s}}\ \lambda_{solution}\left[1 - e^{-d/\lambda_{solution}}\right] \qquad (S2)$$

where $n_{\mathrm{w}}^{\mathrm{m}}$ and $n_{\mathrm{w}}^{\mathrm{s}}$ are the number density of oxygen atoms and $\sigma_{\mathrm{w}}^{\mathrm{m}}$ and $\sigma_{\mathrm{w}}^{\mathrm{s}}$ are the photoionization cross-section of sorbed water inside the membrane and oxygen in the solution layer, respectively. Water content inside the solution-equilibrated membrane is estimated from the number of sorbed water molecules per sulfonate group for CR-61 membranes exposed to water vapor (hydrated conditions) before the dip and pull procedure. In order to do that, the ratio of O *1s* peak areas of LPW to sulfonate components is multiplied by a stoichiometric factor of 3, to be representative of the number of functional groups. $MgCl_2$ equilibrated CR-61 membranes showed higher water content inside the membrane, similar to what we observed in our previous study on polyelectrolytes having the same chemistry.[3]

In addition, electron inelastic mean free paths (IMFPs) in the aqueous salt solution and the membrane are estimated from the Tanuma-Powell-Penn (TPP-2M) algorithm and the modified Bethe equation[4,5]. For the membrane, the polystyrene sulfonate monomer is used to estimate the related parameters.[5] By substituting the values of parameters listed in **Supplementary Table 2** and the relative intensities of the solution and membrane related O *1s* (LPW and SO_3^- components) XPS peaks (given in **Supplementary Table 3 and 4**) into the above equations, we estimated the thickness of the liquid layer to be around ~17-22 nm.

Although the relative intensities of the solution and membrane related O *1s* peaks show fluctuations from spot to spot, they are largely very similar in terms of the actual thickness of the film. For the lowest ratio obtained for NaCl equilibrated CR-61 system (i.e., relative intensity is equal to 7.0) the thickness of liquid layer is calculated as 17 nm, whereas for the largest ratio

obtained for NaCl equilibrated CR-61 system (i.e., relative intensity = 9.8) the calculated thickness of the liquid layer is 20 nm. Similarly, for the lowest ratio obtained for $MgCl_2$ equilibrated CR-61 system, the calculated thickness of the liquid layer is 17 nm, vs. 21 nm for the largest ratio obtained for $MgCl_2$ equilibrated CR-61.Thus, although the relative intensities of the solution and membrane related O *1s* (LPW and SO_3^- components) XPS peaks of the $MgCl_2$ equilibrated membrane are higher (see **Supplementary Table 3 and 4**), the thickness of the liquid layer was estimated to be similar to those equilibrated with NaCl.

With an estimated liquid layer ~17-21 nm thickness, the intensity of the O *1s* peak for the SO_3^- component decreases by ~88% of its initial (dry) value due to attenuation through the liquid layer, which requires more collection time to get sufficient signal to noise of the spectra.

Supplementary Note 2.

Numerical Simulation of Potential Profile at Membrane/Solution Interface

To simulate the electrical potential distribution across a charged membrane in equilibrium with an electrolyte solution, we used a model previously introduced by Ohshima *et al.*[8]. It is assumed that our ion permeable membrane has uniformly distributed, fixed negative charges, and the membrane is in equilibrium with a large volume of a symmetrical electrolyte solution of concentration C_g^s and valence z. We select the x-axis to be the direction normal to the membrane surface, so the plane at x = 0 coincides with the membrane/solution interface (see **Supplementary Fig. 4**).

We assume that the electrical potential at position x in the solution and membrane regions satisfies the Poisson-Boltzmann equation, as follows:

For the solution side, where x<0:

$$\frac{d^2\Psi}{dx^2} = \frac{2zeC_g^s}{\epsilon_r\epsilon_0} sinh\frac{ze\Psi}{kT} \tag{S3}$$

For the membrane side, where x>0:

$$\frac{d^2\Psi}{dx^2} = \frac{2zeC_g^s}{\epsilon_r'\epsilon_0} sinh\frac{z\Psi}{kT} + \frac{eC_g^m}{\epsilon_r'\epsilon_0} \tag{S4}$$

where *T* is temperature, *z* is the charge, *k* is the Boltzmann constant, *e* is the elementary electric charge, C_g^s and C_g^m are the concentration of counter-ions in the solution and the membrane phase, respectively, ϵ_r is the relative permittivity of the solution, and ϵ_r' is the relative permittivity of the membrane.

By introducing a dimensionless potential, y=$\frac{ze\psi}{kT}$, equations (S3) and (S4) are further reduced to:

$$\frac{d^2\Psi}{dx^2} = \frac{2zeC_g^s}{\epsilon_r\epsilon_0} sinh\, y \tag{S5}$$

$$\frac{d^2\Psi}{dx^2} = \frac{2zeC_g^s}{\epsilon_r'\epsilon_0}(sinh\, y + \frac{C_g^m}{zeC_g^s}) \tag{S6}$$

Boundary conditions for our experimental system are:

1) The electrical potential of the bulk solution is equal to zero:

$$y(x) \xrightarrow{x \to -\infty} 0 \text{ and } \frac{d^2\Psi}{dx^2} = 0$$

2) The electrical potential of bulk membranes is equal to the Donnan potential:

$$\frac{d^2\Psi}{dx^2} = 0 \text{ at } x = +\infty \text{ and } y(\infty) = -\,\text{arcsinh}\frac{C_g^m}{2zC_g^s}$$

3) The electrical potential is continuous at the interface:

$$\epsilon_r \left.\frac{dy}{dx}\right|_{-0} = \epsilon'_r \left.\frac{dy}{dx}\right|_{+0}$$

Using reduced equations (S5 and S6) and the boundary conditions above, the ordinary differential equations (ODEs) for both the membrane and the solution side are solved using SciPy's[9] initial value problem solver with the RK45 method.[10] Due to the monotonicity of the model, any given pair of possible numerical solutions on both sides of the interface should satisfy assumption (3) at just one unique set of x values. **Supplementary Fig. 5** shows the simulated potential distribution at the NaCl solution/membrane interface via the classic Donnan model at our experimental conditions, where $T = 298\,K, z = 1$, $\epsilon_r = 78.5$ $\epsilon_r' = 42$, $C_g^m = 3.21$ M and $C_g^s = 0.001 -$ 1M. As gathered from Supplementary Fig.3., y(x) exhibits a Gouy-Chapman type diffuse layer decay for x < 0.

Supplementary Note 3.

Simulation XP Spectra from the Potential Distribution at the Membrane/Solution Interface

After numerically solving the related Poisson-Boltzmann equations to simulate the potential distribution at the membrane/solution interface, we used Python to simulate the S *1s* and O *1s* core level XPS spectra. The liquid phase water (LPW) and membrane phase related-core levels (S *1s*) spectra are described as the convolution of the binding energy shifted spectra of the elements as a function of their position within the potential drop with respect to the membrane/solution interface.[6]

The starting spectra were modelled as a Gaussian function. Three pieces of information are needed to numerically simulate the starting spectra: i) the binding energy (BE), which determines the center of each individual peak; ii) the Gaussian broadening, which is obtained from the experimental full width at half maximum (FWHM); and iii) peak area, which is determined from the number density of each element, integrated over the exponential escape probability of the photoelectron intensity (Beer-Lambert Law).[11,12] Binding energy differences between various peaks and the related FWHM values were obtained from our previous study of the same polymer.[3] Number densities for each element used in the simulations are given in **Supplementary Table 4.**

S *1s* and O *1s* core level XPS peaks were obtained by summation of individual spectra generated every 0.1 Å steps within the potential drop. The binding energy location of each individual spectrum shifted 1 V:1 eV, following the electrical potential profile, as a function of distance from the interface. In addition, the exponential decrease in intensity for each individual spectrum as a function of depth is modelled by the Beer–Lambert law:

$$\frac{I_Z}{I_0} = e^{-\frac{z}{\lambda}} \tag{S7}$$

where z is the probing depth of the membrane with respect to the interface, and λ is the inelastic-

mean-free-path (IMFP) of the photoelectron. The Tanuma-Powell-Penn (TPP-2M) algorithm and the modified Bethe equation are used to obtain IMFP of photoelectrons, generated by an incoming photon of 4.0 keV energy, in the aqueous salt solution and membrane[4,5]. The membrane's related parameters are estimated from the polystyrene sulfonate monomer. λ is estimated to be 4.1 nm for the S *1s* (BE ≅ 2477 eV) and 9.5 nm for the O *1s* (BE ≅ 533.11 eV) escaping photoelectron.

Supplementary Note 4.

Binding Energy Calibration and Effect of EDL Thickness on the Measured Binding Energy of Solution Related Core Levels

At thermodynamic equilibrium and in the absence of any external electrical potential, the potential of the bulk electrolyte should be the same, and equal to zero, independent of the solution concentration. The electrical potential of the CR-61 membrane, on the other hand, changes relative to that of the bulk electrolyte. To follow the electrical potential changes in the membrane, binding energy is calibrated by aligning bulk electrolyte using the LPW peaks in the O *1s* region. For high salt concentrations, where the EDL thickness is small, the contribution from molecules within the bulk electrolyte dominates the LPW signal in the O *1s* region, leading to no spectral shift or broadening of the photoelectron peaks, but when the EDL thickness approaches the experimental probing depth (at low concentration), electrolyte related core level peaks undergo BE shifts and asymmetric spectral broadening (**Supplementary Fig. 3)**. To account for this effect, which is especially evident at lower electrolyte concentrations, we simulated the potential distribution at a charged membrane/solution interface by solving the nonlinear Poisson-Boltzmann equation for the exterior and interior regions of the membrane **(Supplementary Fig. 5)** The EDL thicknesses for various external concentrations, estimated from the commonly used Debye-Huckel Theory[13] (linearized Poisson-Boltzmann equation) and the Poisson-Boltzmann simulations **(Supplementary Note 2)**, are given in **Supplementary Table 7.** Electrical double layer thickness from the numerical solutions of Poisson-Boltzmann equation are obtained from the overall length of Gouy-Chapman type diffuse layer in **Supplementary Fig. 5.** As expected, the Debye length underestimates the EDL width. Corresponding BE shifts in LPW were estimated by simulating XP spectra from the potential distributions within the EDL region. **Supplementary Table 8** shows the

relative shifts in the binding energy of LPW obtained from simulations. For external NaCl concentrations higher than 0.01 M, the effect of the EDL on the binding energy of the LPW O *1s* peak became negligible, within our experimental uncertainties. Similarly, for Donnan potential measurements on CR-61 membranes equilibrated with $MgCl_2$, the effect of the EDL on binding energy of the LPW peak is also negligible, due to an overall decrease in the potential drop. For this reason, experimentally measured LPW binding energies for various $MgCl_2$ solution concentrations have been considered to originate only from the bulk liquid, and calibration was done without further corrections.

Supplementary Note 5.

Binding energy shifts of other core levels

In theory, the Donnan potential of CR-61 membranes equilibrated with aqueous salt solutions can also be assessed from the binding energy shifts of other membrane related core levels (i.e., O *1s*-SO_3 and C *1s*). **Supplementary Table 6** shows the binding energies of fitted XP spectra for O *1s*-LPW, O *1s*-GPW, O *1s*-SO_3, S *1s*-SO_3 and Na/Mg *1s*. C1s core level spectra are also collected during the experiments. However, the C1s region consists of at least 5 different chemistries including aromatic, aliphatic, and C-SO_3 from the membrane. Additionally, the C *1s* region is susceptible to further changes upon exposure to water (in the form of adventitious carbon and other carbonaceous species, i.e., C-O and C=O). Deconvolution of this peak is challenging since the many individual contributions forming the C *1s* peak are not well resolved. We also could not constrain the area of any membrane components during the fitting procedure, due to the unknown percentage of cross linker in the membrane assembly. This made the peak-fitting process challenging and following such small binding energy differences in the region unreliable. That is why we have not presented the binding energies of C *1s* chemistries here. Representative C *1s* spectra for each concentration NaCl and $MgCl_2$ are provided in **Supplementary Fig. 7** with the binding energy of possible individual chemical carbon contributions. As can be gathered from the figure, no carbonate species are detected in the C *1s* spectra, which generally appears at binding energies higher than 288.5 eV.[6] This is important especially for this type of experiment as competitive ion sorption by other ions in solution (e.g., carbonates from CO_2 speciation) could introduce error in the Donnan Potential measurement.

As predicted, the binding energy shifts in membrane O *1s* are consistent with those in S *1s* (see **Supplementary Fig. 8 and Table 6**). However, the O *1s* region requires multi-peak fitting, which introduces additional uncertainties and increases the error bars of the measurement. In addition, due to the nature of this experimental technique, membrane related core level photoelectrons are attenuated through both the liquid and the gas phase. Low binding energy peaks shows relatively low photoelectron intensities due to their low cross sections. Considering these factors, in the main text, we chose a single component, high binding energy S *1s* core level to measure the Donnan potential, leading to smaller error bars and more precise measurements.

We would like to note that the counter-ion binding energies show a different behavior. Contrary to our observations in the immobile membrane peaks, no clear concentration dependence is observed in the counter-ion related binding energy of NaCl- and $MgCl_2$-equilibrated CR-61 (see **Supplementary Fig. 9** and **10**). This behavior may arise from the weak interaction with the membrane sulfonate charges and/or the strong hydration shell of counter-ions. We are currently devoting time and effort to better understand this phenomenon, and it will be the focus of a future manuscript.

In addition to counter-ions, co-ion (Cl *1s*) core level spectra were also collected during the experiments, but no peak was observed for electrolyte concentrations lower than 1 M. Considering the nature of ion exchange membranes, where co-ion concentration inside the membrane is very low compare to the counter-ion concentration, which is dictated by the number of fixed charges (i.e., for CR-61 = 3.2 M), we believe that observed counter-ion specific peaks were coming mainly from the ions inside the membrane at lower salt concentration. This sorption behavior is supported by quantitative analysis and numerical simulations on Na *1s*/S *1s* and Mg *1s*/S *1s* peak area ratios given in **Supplementary Note 6.** As predicted, peak area ratios of counterion to membrane related

core levels do not show any obvious trend or change at lower concentration. On the other hand, at 0.3 M salt concentration, Na *1s*/S *1s* and Mg *1s*/S *1s* area ratios starts to increase due to the additional contribution from detection of counter-ions in the liquid layer.

Since the detected Mg *1s* and Na *1s* regions at 1M solution concentration are a convolution of peaks coming from ions in both the membrane and liquid phase, the binding energy values at 1 M external solution concentration are excluded from the plot given in **Supplementary Fig. 10**. Unfortunately, it was not possible to deconvolute the solution phase ions from the ions inside the membrane because of small binding energy/electrical potential differences between them, which were not enough to form any peak separation or broadening. However, the small drop in overall Mg *1s* BE at 1 M solution concentration (**Supplementary Table 6.** and **Fig. 9b**) may arise from an additional contribution from the liquid layer which was not detectable at lower concentrations.

In addition, no significant trend for binding energies of O *1s*-GPW peak was observed with respect to the external solution concentration.

Supplementary Note 6.

Numerical simulation of the photoelectron intensity

Since we established that the thicknesses of the liquid layer on the CR-61 ion exchange membrane with various salt concentrations are similar in **Supplementary Note 1**, the sorption of counter-ions in CR-61 membranes over a range of external solution concentrations can be quantified from the relative intensities of the counter-ion peaks (Na *1s* or Mg *1s*) and membrane related S *1s*. The simulations are built adopting a layered structure, where the membrane is simply buried underneath a salt solution layer of thickness d film. Accordingly, the intensity of the Na *1s* and Mg *1s* peaks at various external salt concentrations is obtained by integrating over the exponential escape probability as follows:

$$I_{Mg} = S_{Mg}\, n_{\mathrm{Mg}}^{\mathrm{m}}\, \lambda_{memb}\, e^{-d/\lambda_{solution}} + S_{Mg}\, n_{\mathrm{Mg}}^{\mathrm{s}}\, \lambda_{solution}\left[1 - e^{-d/\lambda_{solution}}\right] \quad \text{(S8)}$$

$$I_{Na} = S_{Na}\, n_{\mathrm{Na}}^{\mathrm{m}}\, \lambda_{memb}\, e^{-d/\lambda_{solution}} + S_{Na}\, n_{\mathrm{Na}}^{\mathrm{s}}\, \lambda_{solution}\left[1 - e^{-d/\lambda_{solution}}\right] \quad \text{(S9)}$$

where $n_{\mathrm{Mg}}^{\mathrm{m}}$, $n_{\mathrm{Na}}^{\mathrm{m}}$ and $n_{\mathrm{Na}}^{\mathrm{s}}$, $n_{\mathrm{Mg}}^{\mathrm{s}}$ are the number density of counter-ions inside the membrane and in the solution phase, respectively. The number density of counter-ions inside the membrane is estimated from the concentration of fixed charges in CR-61. λ_{memb} and $\lambda_{solution}$ are the IMFPs in the membrane and solution, respectively. The sensitivity constants, S_{Mg} and S_{Na}, are instrumental parameters which depend on the photoionization cross-section of elements at a given X-Ray energy, X-ray flux at a given X-ray energy, the orbital specific asymmetry, and the spectrometer efficiency for a given kinetic energy (KE). S must be taken into an account when quantifying photoelectron peaks with different KE.

Similarly, the intensity of the S *1s* peaks of a CR-61 membrane underneath a liquid layer of thickness d, over the entire concentration range probed, is obtained from:

$$I_S = S_S\, n_S^{\mathrm{m}}\, \lambda_{memb}\, e^{-d/\lambda_{solution}} \qquad \text{(S10)}$$

By substituting the values of IMFPs listed in **Supplementary Table 9,** the relative intensities of the Na *1s* or Mg *1s* and S *1s* XPS peaks are simulated over the entire salt concentration range probed. (**Supplementary Fig. 11**) The simulated intensity ratios agree with experimental observations. As expected, peak area ratios of counterion to membrane related core levels do not show any obvious trend or change at lower concentration. On the other hand, Na *1s*/S *1s* and Mg *1s*/S *1s* area ratios start to increase at high concentrations due to additional contribution from detection of counter-ions in the liquid layer. It needs to be highlighted that these intensity simulations were performed using homogeneous and well-defined layered structures and interfaces as an approximation of the real configuration, where most likely concentration gradients and mixed regions exist. In addition, it was previously established that the surface composition of the salt solutions are enhanced in the halide anion concentration (and thus attenuation of the cation) compared with the bulk of the solution at the liquid/vapor interface.[14,15] Given these assumptions there is reasonable agreement between our experimental and simulated data, which reveal a similar trend of the primary detectable spectra contributions.

Supplementary Note 7.

Experimental uncertainties and statistical significance

Since our approach to directly measuring the Donnan potential at the membrane/solution interface is based on following small binding energy shifts of membrane related core level XP spectra, we utilized a number of statistical methods to estimate the experimental uncertainties, determining outlier data points and evaluating statistical significance of our data. As mentioned in the main text, the error bars used to represent experimental uncertainty are determined from the standard deviation of repeated measurements of 4-5 different positions in each case.

In addition, we employed the statistical "Q test" to determine if a single data value is an outlier in a distinct sample size. This test essentially calculates the ratio between the putative outlier's distance from its nearest neighbor and the range of values. Upon comparing the calculated Q to the theoretical Q, one very large (2477.12 eV S *1s* binding energy for CR-61 equilibrated with 0.1M NaCl) and one very small (2476.93 eV S *1s* binding energy for CR-61 equilibrated with 0.1M NaCl) value were rejected from the data set by the Q-test with 95% confidence.

To demonstrate that the slopes of linear fit to our experimentally measured binding energies of S *1s* peak are statistically different from zero and from each other for NaCl and $MgCl_2$ equilibrated CR-61 membranes, we ran a statistical t-test (inferential statistical test used to determine if there is a significant difference between the means of two groups) and an ANOVA (Analysis of Variance) analysis using Origin software. Further information regarding these statistical analyses can be found elsewhere[16]. The data in **Fig. 3** are presented using the raw data in **Supplementary Fig. 14,** and the related parameters from the statistical analysis are given in **Supplementary Tables 10** and **11** for NaCl and $MgCl_2$, respectively. Since the t and F values exceed the critical values of t[16] and F[16] at 95% confidence level for specific degrees of freedom (DF), we conclude that the slopes are

statistically different from zero and from each other.

Supplementary Note 8.

Model Predictions of the Donnan Potential

As discussed in the main text, the presence of fixed charge groups inherently leads to an unequal distribution of counter-ions and co-ions in IEMs. This phenomenon generates an electrical potential at the membrane/solution interface, referred to as the Donnan potential[17] (Ψ_{Donnan}), that acts to attract counter-ions into the membrane and restrict co-ions from entering the membrane[18]. Following a standard thermodynamic treatment, Ψ_{Donnan} is defined as follows[17,18]:

$$\Psi_{\text{Donnan}} = \frac{RT}{z_i F} ln \frac{\gamma_i^s C_i^s}{\gamma_i^m C_i^m} \quad \text{(S11)}$$

where R is the universal gas constant, T is the temperature, F is Faraday's constant, z_i is the valence of ion i, C_i^s is the concentration of ion i in the external solution, C_i^m is the concentration of ion i in the membrane (mol/L sorbed water), γ_i^s is the activity coefficient of ion i in the external solution, and γ_i^m is the activity coefficient of ion i in the membrane. Thus, Ψ_{Donnan} depends strongly on ion valence and the difference between an ion's activity in the membrane and solution phases. For a given IEM, the fixed charge concentration of the membrane, the counter-ion identity, and the external salt concentration should largely govern Ψ_{Donnan}. Knowledge of individual ion concentrations and activity coefficients in each phase permits calculation of Ψ_{Donnan}.

Using thermodynamic models based on the Donnan and Manning theories[1,19], we calculated theoretical predictions of Ψ_{Donnan} for CR-61 membranes equilibrated with NaCl and $MgCl_2$. More details regarding these analytical models are provided elsewhere[20]. The classic Donnan expression describing equilibrium of ions between the solution and membrane phases is[18]:

$$\left(C_g^m\right)^{\nu_g}\left(\gamma_g^m\right)^{\nu_g}(C_c^m)^{\nu_c}(\gamma_c^m)^{\nu_c} = \left(C_g^s\right)^{\nu_g}\left(\gamma_g^s\right)^{\nu_g}(C_c^s)^{\nu_c}(\gamma_c^s)^{\nu_c} \quad \text{(S12)}$$

where g, c, m, s, and ν refer to counter-ions, co-ions, the membrane, the solution, and the stoichiometric coefficient of an ion, respectively. Incorporating electroneutrality conditions into Donnan's model yields an equation that can be solved for C_c^m, based on the fixed charge concentration of the membrane, C_A^m, and expressions for γ_i in each phase. Electroneutrality in a charged membrane is expressed as[18,19]:

$$\sum_i z_i C_i^m + \omega C_A^m = 0 \quad \text{(S13)}$$

where ω is the valence of the fixed charge groups (e.g., –1 for CR-61). In Donnan's original theory (the classic Donnan model), ion activity coefficients are eliminated from equation **S11** by assuming either ion activity coefficients of unity (i.e., thermodynamic ideality) or equality of ion activity coefficients:

$$\left(\gamma_g^m\right)^{\nu_g}(\gamma_c^m)^{\nu_c} = \left(\gamma_g^s\right)^{\nu_g}(\gamma_c^s)^{\nu_c} \quad \text{(S14)}$$

C_A^m values were calculated from previously reported data for the ion exchange capacity (IEC) and water uptake (w_u) of CR-61. The following expression relates these parameters[19]:

$$\text{IEC} = \frac{C_A^m w_u}{\rho_w} \quad \text{(S15)}$$

where ρ_w is the density of water. Values for NaCl- and $MgCl_2$- equilibrated CR-61 were calculated using data from Kamcev *et al.*[19] and Galizia *et al.*[2], respectively. **Supplementary Table 12** presents values of C_g^m at a given external salt concentration predicted using the classic Donnan model (i.e., eliminating ion activity coefficients).

Although straightforward, the classic Donnan model is often criticized for its poor quantitative predictions of ion sorption in IEMs.[21] Moreover, the assumption taken regarding activity

coefficient values being unity is inconsistent with experiments and models for aqueous solutions[22] and polyelectrolytes[1,20 23]. Kamcev *et al.*[19] previously incorporated ion activity coefficient expressions into equation S9, validating this approach as a good predictor of ion sorption in several IEMs, including CR-61. This framework uses Manning's counter-ion condensation theory[20], originally developed for polyelectrolytes, to describe membrane phase non-idealities and applies the Pitzer model[24] to describe solution phase non-idealities. To allow calculation of ion activity coefficients,[20] Manning derived an expression for the excess free energy of a polyelectrolyte with added salt, accounting for the electrostatics that lead to the condensation of counter-ions near fixed charge groups. Manning's theory requires a single parameter, ξ, which is defined as[20]:

$$\xi = \frac{\lambda_b}{b} = \frac{e^2}{4\pi\varepsilon_0\varepsilon kTb} \tag{S16}$$

where λ_b is the Bjerrum length, b is the average distance between fixed charge groups, e is the protonic charge, ε_0 is the vacuum permittivity, ε is the dielectric constant of the solution, k is Bolztmann's constant, and T is absolute temperature. ξ represents a dimensionless polyelectrolyte charge density. For an IEM, ξ can be estimated by using the ion exchange capacity and dielectric constant of the hydrated membrane. Kamcev *et al.*[1] estimated a ξ value of 1.83 for CR-61. For the case of counter-ion condensation, Manning's expressions for γ_g^m and γ_c^m are[19]:

$$\gamma_g^m = \left(\frac{\frac{1}{z_g\xi}X + z_g\nu_g}{X + z_g\nu_g}\right)\exp\left(-\frac{\frac{X}{2}}{X + z_g\nu_g\xi(\nu_g + \nu_c)}\right) \tag{S17}$$

$$\gamma_c^m = \exp\left(-\frac{\frac{X}{2}\left(\frac{z_c}{z_g}\right)^2}{X + z_g\nu_g\xi(\nu_g + \nu_c)}\right) \tag{S18}$$

where X is defined as C_A^m/C_s^m, and where C_s^m is the mobile salt concentration in the membrane and is stoichiometrically related to the co-ion concentration in the membrane:

$$C_s^m = \frac{C_c^m}{\nu_c} \quad \text{(S19)}$$

Equations **S12, S13**, and **S17-S19** were solved numerically to predict C_i^m and γ_i^m in CR-61 equilibrated with 0.001-1 M NaCl and $MgCl_2$, using known C_A^m values and ξ = 1.83[19]. Prior work has shown good agreement between these predictions and experimental ion sorption data from CR-61[19,2]. The Pitzer model was used to calculate the mean ion activity coefficient, $\gamma_\pm^s$, in the external solution, due to the model's excellent agreement with $\gamma_\pm^s$ data over a broad range of ionic strength[24]. $\gamma_\pm^s$ is defined as the geometric mean of the individual ion activity coefficients[22]:

$$\gamma_\pm^s = \sqrt[\nu_+ + \nu_-]{(\gamma_+^s)^{\nu_+}(\gamma_-^s)^{\nu_-}} \quad \text{(S20)}$$

Using the predicted values of C_g^m and γ_g^m from the approach outlined above, theoretical Ψ_{Donnan} values were calculated via equation S8. Because the Pitzer model is expressed in terms of $\gamma_\pm^s$, values of γ_g^s for NaCl and $MgCl_2$ at a given ionic strength were approximated using $\gamma_\pm^s$ values for KCl from the Pitzer model at the same ionic strength. Under the assumption that γ_i^s for K^+ and Cl^- are equal, the activity coefficient of Cl^- at a given ionic strength is known and can be used to estimate the activity coefficients of Na^+ and Mg^{2+} in NaCl and $MgCl_2$ at the same ionic strength (**Eq. S20**). Although crude, this estimation is consistent with some aqueous electrolyte data and studies in the literature.[22]

Predicted values, using the Manning/Donnan model, of C_g^m and γ_g^m at a given external NaCl and $MgCl_2$ salt concentration are presented in **Supplementary Table 14** and **Supplementary Table 15**, respectively.

Predicted values of Ψ_{Donnan} for CR-61 using the classical Donnan model are presented in **Supplementary Table 13.**

Supplementary Note 9.

Comparison between experimental data and classical Donnan model

Supplementary Fig. 15a and 15b compare the experimental and classical Donnan model predicted Donnan potential values as a function of external NaCl and $MgCl_2$ solution concentrations, respectively. To assess the agreement between the experimental values and the theoretical model, we calculated the absolute difference from the linear fit of the experimental data. For NaCl equilibrated CR-61 (**Supplementary Fig. 15a**), agreement between the classic Donnan model and experimental data is rather poor at low external solution concentrations. As the external salt concentration increases, agreement between the theoretical model and experimental measurements improve. The inability of the classic Donnan model to accurately predict the Donnan potential arises, at least in part, from strong electrostatic interactions between fixed charge groups and counter-ions, leading to the condensation of counter-ions on the polymer backbone. This phenomenon results in highly non-ideal thermodynamic behavior, especially at low electrolyte concentrations. In contrast, when an IEM is equilibrated with a highly concentrated solution, the fixed charge groups are electrostatically screened by sorbed salt, and the thermodynamic environments of the solution and membrane phases become more similar. In this way, the ion activity coefficients in the membrane and in the external electrolyte solution also become closer to one another, such that the classic Donnan model can provide a reasonable prediction of the Donnan potential in CR-61 equilibrated with 1 M NaCl.

For $MgCl_2$ equilibrated CR-61 (**Supplementary Fig. 15b**), predictions using the classic Donnan model correlate reasonably well with the experimental measurements. For $MgCl_2$ equilibrated CR-61, fewer counter-ions are present in the membrane at any given external salt concentration than for NaCl, leading to relatively low Donnan potential values. Moreover, previous studies of CR-61

have shown that activity coefficients in the membrane are more similar to activity coefficients in the external solution for $CaCl_2$ equilibrated CR-61 than for NaCl equilibrated CR-61, particularly at higher external salt concentrations.[25] The ability of the classic Donnan model to reasonably predict the Donnan potential, within the error of our measurements, arises from the similar behavior of $MgCl_2$ equilibrated to that of $CaCl_2$.

Supplementary References

1 Kamcev, J., Paul, D. R. & Freeman, B. D. Ion activity coefficients in ion exchange polymers: Applicability of manning's counterion condensation theory. *Macromolecules* **48**, 8011-8024 (2015).

2 Galizia, M., Benedetti, F. M., Paul, D. R. & Freeman, B. D. Monovalent and divalent ion sorption in a cation exchange membrane based on cross-linked poly (p-styrene sulfonate-co-divinylbenzene). *J. Membr. Sci.* **535**, 132-142 (2017).

3 Gokturk, P. A., Barry, M., Segalman, R. & Crumlin, E. J. Directly probing polymer thin film chemistry and counterion influence on water sorption. *ACS Appl. Poly. Mater.* **2**, 4752-4761 (2020).

4 Tanuma, S., Powell, C. J. & Penn, D. R. Calculation of electron inelastic mean free paths (imfps) vii. Reliability of the tpp-2m imfp predictive equation. *Surf. Interface Anal.* **35**, 268-275 (2003).

5 Tanuma, S., Powell, C. J. & Penn, D. R. Calculations of electron inelastic mean free paths. V. Data for 14 organic compounds over the 50–2000 ev range. *Surf. Interface Anal.* **21**, 165-176 (1994).

6 Favaro, M. *et al.* Unravelling the electrochemical double layer by direct probing of the solid/liquid interface. *Nat. Commun.* **7**, 12695 (2016).

7 Beamson, G. & Briggs, D. *High resolution xps of organic polymers: The scienta esca300 database* (Wiley, Chichester, 1992).

8 Ohshima, H. & Ohki, S. Donnan potential and surface potential of a charged membrane. *Biophys. J.* **47**, 673-678 (1985).

9 Virtanen, P. *et al.* Scipy 1.0: Fundamental algorithms for scientific computing in python.

Nature Methods **17**, 261-272 (2020).

10 Dormand, J. R. & Prince, P. J. A family of embedded runge-kutta formulae. Journal of Computational and Applied Mathematics **6**, 19-26 (1980).

11 Cumpson, P. J. The thickogram: A method for easy film thickness measurement in xps. *Surf. Interface Anal.* **29**, 403-406 (2000).

12 Strohmeier, B. R. An esca method for determining the oxide thickness on aluminum alloys. *Surf. Interface Anal.* **15**, 51-56 (1990).

13 Bard, A. J. & Faulkner, L. R. Electrochemical methods: Fundamentals and applications (Wiley, 2000).

14 Ghosal, S. *et al.* Electron spectroscopy of aqueous solution interfaces reveals surface enhancement of halides. *Science* **307**, 563-566 (2005).

15 Ghosal, S. *et al.* Ion partitioning at the liquid/vapor interface of a multicomponent alkali halide solution: A model for aqueous sea salt aerosols. The Journal of Physical Chemistry A **112**, 12378-12384 (2008).

16 Skoog, D. A., Holler, F. J., Crouch, S. R. & West, D. M. *Fundamentals of analytical chemistry Ch. 7* (Cengage, Belmont, 2014).

17 Donnan, F. G. The theory of membrane equilibria. *Chem. Rev.* **1**, 73-90 (1924).

18 Helfferich, F. G. *Ion Exchange Ch. 8* (Dover, New York, 1995).

19 Kamcev, J. et al. Partitioning of mobile ions between ion exchange polymers and aqueous salt solutions: Importance of counter-ion condensation. *Phys. Chem. Chem. Phys*. **18**, 6021-6031 (2016).

20 Manning, G. S. Limiting laws and counterion condensation in polyelectrolyte solutions i. Colligative properties. *J. Chem. Phys.* **51**, 924-933 (1969).

21 Glueckauf, E., Watts, R. E. & Spence, R. The donnan law and its application to ion exchanger polymers. *Proc. R. Soc. A Math. Phys. Eng. Sci.* **268**, 339-349 (1962).

22 Robinson, R. A. & Stokes, R. H. Electrolyte solutions (Courier Corporation, 2002).

23 Yu, Y., Li, Y., Hossain, N. & Chen, C.-C. Nonrandom two-liquid activity coefficient model for aqueous polyelectrolyte solutions. *Fluid Phase Equilib.* **497**, 1-9 (2019).

24 Pitzer, K. S. Thermodynamics of electrolytes. I. Theoretical basis and general equations. *J. Phys. Chem*. **77**, 268-277 (1973).

25 Galizia, M., Manning, G. S., Paul, D. R. & Freeman, B. D. Ion partitioning between brines and ion exchange polymers. *Polymer* **165**, 91-100 (2019).
